# Developing community resilience in the face of COVID-19: case study from the Estrie region, Canada

**DOI:** 10.1093/heapro/daae094

**Published:** 2024-08-24

**Authors:** Martine Shareck, Marie Suzanne Badji, Karine Picard, Jean-François Allaire, Philippe Roy, Mélissa Généreux, Julie Lévesque, Émanuèle Lapierre-Fortin

**Affiliations:** Département des sciences de la santé communautaire, Faculté de médecine et des sciences de la santé, Université de Sherbrooke, 3001 12 Ave N, Sherbrooke, Quebec, J1H 5H3, Canada; Centre de Recherche du Centre Hospitalier de l’Université de Sherbrooke, 12e Ave N, Sherbrooke, Quebec, J1H 5N4, Canada; Institut Universitaire de Première Ligne en Santé et Services Sociaux du CIUSSS de l’Estrie-CHUS, 1036 Rue Belvédère S, Sherbrooke, Quebec, J1H 4C4, Canada; Observatoire Estrien du Développement des Communautés, 1820 Rue Galt O, Sherbrooke, Quebec, J1K 1H8, Canada; Observatoire Estrien du Développement des Communautés, 1820 Rue Galt O, Sherbrooke, Quebec, J1K 1H8, Canada; Institut Universitaire de Première Ligne en Santé et Services Sociaux du CIUSSS de l’Estrie-CHUS, 1036 Rue Belvédère S, Sherbrooke, Quebec, J1H 4C4, Canada; Institut Universitaire de Première Ligne en Santé et Services Sociaux du CIUSSS de l’Estrie-CHUS, 1036 Rue Belvédère S, Sherbrooke, Quebec, J1H 4C4, Canada; Ecole de travail social, Université de Sherbrooke, 2500 Bd de l’Université, Sherbrooke, Quebec, J1K 2R1, Canada; Département des sciences de la santé communautaire, Faculté de médecine et des sciences de la santé, Université de Sherbrooke, 3001 12 Ave N, Sherbrooke, Quebec, J1H 5H3, Canada; Institut Universitaire de Première Ligne en Santé et Services Sociaux du CIUSSS de l’Estrie-CHUS, 1036 Rue Belvédère S, Sherbrooke, Quebec, J1H 4C4, Canada; Direction de santé publique, CIUSSS de l’Estrie-CHUS, 300 Rue King E, Sherbrooke, Quebec, J1G 1B1, Canada; Institut national de santé publique du Québec, 945 Av. Wolfe, Quebec City, Quebec, G1V 5B3, Canada; Observatoire Estrien du Développement des Communautés, 1820 Rue Galt O, Sherbrooke, Quebec, J1K 1H8, Canada

**Keywords:** case study, community development, community resilience, COVID-19, crisis, emergency, equity, intersectoral collaboration

## Abstract

The COVID-19 pandemic undeniably impacted population health and several aspects of community organization, including service delivery and social cohesion. Intersectoral collaboration and equity, two key dimensions of community resilience, proved central to an effective and equitable response to the pandemic. Yet the factors that enabled or constrained communities’ capacity to enact intersectoral collaboration and equity-focused action in such times of urgency and uncertainty remain poorly understood. This descriptive qualitative study aimed to (1) describe the processes through which intersectoral collaboration and equity-focused action were deployed during the first wave of COVID-19 and (2) identify factors enabling and constraining these processes. We conducted semi-directed interviews with 35 representatives of the governmental, institutional, and public and third sectors from four municipal regional counties of the Estrie region (Québec, Canada). We coded detailed interview notes following a codebook thematic analysis approach. We identified three processes through which intersectoral collaboration and equity-focused action were deployed: (1) networking; (2) adaptation, creation and innovation; and (3) human-centred action. Examples of levers which supported the deployment of these processes included capitalizing on pre-existing networks, adapting practices and services, and investing in solidarity and mutual aid. The influencing factors we describe represent concrete targets for resilience-building action. Although focused on the COVID-19 pandemic, our findings are relevant to other types of health, social, environmental or economic crises, and may guide health promotion and community development practitioners towards more effective community resilience-building responses.

**Contribution to Health Promotion**

- Community resilience and health promotion share key areas of action, such as intersectoral collaboration and equity.
- Community resilience must be fostered for effective and equitable response to future crises and emergencies such as the climate crisis.
- In our study, networking, adaptation, creation and innovation, and human-centred action were central to an intersectoral and equitable response to COVID-19 in the Estrie region.
- These processes and associated influencing factors apply to other similar crises and emergencies, supporting a health and equity-promoting response.

## INTRODUCTION

The COVID-19 pandemic and associated public health measures implemented to curb the spread of the coronavirus had major impacts on health and health inequity (Public Health Agency of Canada, 2023) as well as on economic vitality, public safety, employment, food security, access to resources and social cohesion at the community level (Public Health Agency of Canada, 2020; Barlow *et al*., 2021). In Québec, Canada, government-imposed confinement was part of such public health measures. Following the declaration of a state of emergency on 13 March 2020, all non-essential activities were temporarily halted, physical distancing and work-from-home measures were implemented, and indoor and outdoor gatherings were prohibited at several timepoints during the first and subsequent waves of COVID-19 (Institut National de Santé Publique du Québec, 2022).

Combined to the increased power granted to the State and the rise of public health as a leader of the health crisis, these types of restrictions forced institutions and organizations to operate in a new and changing context of high uncertainty, with consequences for human relations, management and service provision (Badji *et al*., 2023; Cunningham *et al*., 2023). For example, injunctions to limit physical and social contacts upset the usual communication and collaboration mechanisms between community development actors (Badji *et al*., 2023). They also led to a decline in trust in public health authorities and governments (Schluter *et al*., 2023). By reducing the potential for mobilizing community development and other actors around collective action, these upheavals tested communities’ capacity to exert resilience (Badji *et al*., 2023), i.e. the ability to adapt to an environment characterized by change, uncertainty and surprise, by mobilizing collective resources (Magis, 2010). On a brighter side, they also spurred the development of new ways of thinking and doing collective action to address issues which emerged during, or were exacerbated by, COVID-19 (Badji *et al*., 2023).

Community resilience has been conceptualized as encompassing several dimensions which, depending on the framework one draws upon, include adaptation capacity, community capital, collective action, collaboration, local leadership, development and engagement of community resources, strategic action and learning, and inclusion and equity (Magis, 2010; McCrea *et al*., 2014; O’Sullivan *et al*., 2014; Cafer *et al*., 2019). Each framework is distinct, putting more or less emphasis on key dimensions, but there are also overlaps (McCrea *et al*., 2014). Intersectoral collaboration and equity are two dimensions that are recurringly discussed in the community resilience literature. Intersectoral collaboration involves actors from different sectors (e.g. education, health, employment, housing, social services, municipal services) and different orders (public, private, third sector or civil society) pooling their resources to act collectively to address a complex situation of common interest that they cannot resolve alone (Divay *et al*., 2013). As per equity-focused action, in the COVID-19 context, we defined it as action targeting ‘vulnerable groups’, i.e. people or organizations who found themselves at higher risk of suffering from the crisis or its collateral effects (e.g. job losses, school closures, intimate partner violence) because of shared social characteristics (e.g. precarious employment situation, single-parent family, female gender) (Frohlich and Potvin, 2008). Collective action rooted in intersectoral collaboration is thought to offer greater potential for promoting social and health equity than sectoral action (Institut National de Santé Publique du Québec, 2021), since promoting equity requires action on the social determinants of health such as income, employment, housing, education and neighbourhood environments, which lie outside the direct realm of public health (Raphael *et al*., 2020; Amri *et al*., 2022).

While intersectoral collaboration and equity-focused action appear to be essential to identify and respond effectively to the needs of vulnerable groups in normal times, a question remains: in times of urgency and uncertainty as created by the COVID-19 pandemic, how can communities’ capacity to act intersectorally and in favour of equity be supported to promote their resilience? The present study aimed to (1) identify the processes by which intersectoral collaboration and equity-focused action were deployed during the first wave of COVID-19 and (2) document the factors which facilitated or limited these processes. Understanding this is essential to strengthen communities’ capacity to become more resilient in the face of future similar emergencies (Public Health Agency of Canada, 2023; United Nations Office for Disaster Risk Reduction, n.d.). Furthermore, a focus on equity fills a gap in community resilience research and practice given that despite vulnerable groups being more at risk of suffering from crises and emergencies, they have more difficulty recovering (Norris *et al*., 2008) and their voices have often been excluded from community resilience thinking (Cafer *et al*., 2019).

## METHODS

### Context

The current study was embedded within a larger participatory research project initiated by the Observatoire estrien du développement des communautés (OEDC), a not-for-profit organization whose mission is to support Estrie communities in building capacity for collective action. Following concerns expressed by their members in April 2020 regarding how to respond to COVID-19 and its collateral impacts, OEDC set out to draft an intersectoral depiction of the local and regional response to COVID-19 in the Estrie region, and guide communities in developing or reinforcing their resilience. In the first phases of this larger project, intersectoral collaboration and equity stood out as being of particular interest to communities, but they struggled with implementing concrete actions. This observation spurred the present study.

### Study design and case selection

This descriptive qualitative study was conducted in the Estrie region which covers an area of 12 483 km^2^ in the south-eastern part of Québec, Canada, and has an estimated population of 507 208 inhabitants [Institut de la statistique du Québec (ISQ), 2022]. It encompasses nine municipal regional counties (MRCs), eight of which took part in the larger project. Here, we selected four MRC territories based on three diversification criteria to allow for identifying similarities and differences: geography (rural vs urban context), population age distribution (Institut de la statistique du Québec (ISQ), 2022; Statistics Canada, 2022), and previous experience with large-scale economic or social crises. We also took into consideration the quality and depth of interview material (e.g. some interviews were relatively succinct and superficial). Cases included vast, sparsely populated areas as well as the main urban centre, the city of Sherbrooke. Two cases had previously experienced crises and subsequently deployed collective initiatives to recover and rebuild physically, socially and economically (Généreux *et al*., 2020; OEDC, 2020). The Sherbrooke, des Sources, Granit and Haut-Saint-François MRCs were selected as cases and are described in Table 1.

**Table 1:** Main characteristics of the four selected cases

Characteristic	Sherbrooke	Des Sources	Granit	Haut-Saint-François
Urbanicity	Urban	Rural	Rural	Rural
Land area (Statistics Canada, 2022)	353 km^2^	785 km^2^	2732 km^2^	2270 km^2^
Population size (Institut de la statistique du Québec (ISQ), 2022)	175 684	14 732	22 112	23 672
Percentage 65+ years (Institut de la statistique du Québec (ISQ), 2022)	21.4%	29.9%	28.3%	23.3%
Noteworthy contextual elements	Historically strong anchoring in culture of cooperation and concertation.	2012 closing of asbestos and magnesium mine led to the loss of approximately 500 jobs.	2013 derailment of oil-carrying train destroyed Lac Mégantic town centre and killed 47 people.	Comprehensive and integrated development approach adopted in 2015.

### Data collection

We conducted semi-directed individual phone or video interviews in July and August 2020 with 8–10 individuals in each MRC. The study’s advisory board—composed of public health and social work researchers, the OEDC’s executive director, and representatives of the regional social and health services, provincial public health, and regional political sector—identified potential participants. These were representatives of the governmental, institutional or public and third sectors. They worked in the regional, municipal, health, education, economy, social and cultural fields, and had extensive knowledge of, and local involvement in, their MRC territory. Of the 35 participants we interviewed across the four selected MRCs, 8 were community development professionals, 15 were administrators or managers at the municipal level (*n* = 2), in economic development organizations (*n* = 2) and community organizations (*n* = 11), 5 were MRC and city directors, and 7 were elected officials. The interview guide covered the overall response to the first wave of COVID-19 (February 27–July 2020) in participants’ territory, key community resilience dimensions and main learnings. Lasting approximately 60 minutes, interviews were digitally recorded. The interviewer took detailed notes which were subsequently amended by two research assistants upon listening to the recordings. Participants provided informed consent prior to interviews. Ethical approval was obtained from the CIUSSS de l’Estrie-CHUS [2021-3839].

### Data analysis

We performed a thematic codebook analysis of detailed interview notes as per Braun and Clarke (Braun and Clarke, 2006) with NVivo 12 software. The analysis consisted in five phases leading to a final report and manuscript: (1) familiarizing oneself with the data; (2) generating initial codes; (3) searching for themes; (4) reviewing themes; and (5) defining and naming themes. The codebook drew on the research objectives, interview guide and analytical grid used in the larger project which centred around community strengths and weaknesses, pre-existing social infrastructure and how it supported (or limited) intersectoral collaboration and equity-focused action, and how communities identified vulnerable groups and their needs. The codebook structure allowed for including codes emerging inductively. Two research assistants independently coded interview notes. Codes were organized into categories to define key processes deployed for intersectoral collaboration and equity-focused action, and to identify facilitators and barriers to process deployment.

### Criteria for quality

As part of the larger project, we held participatory workshops with study participants and additional key actors from each MRC territory to validate preliminary results (Fall 2020), facilitated two webinars on intersectoral collaboration and equity (Spring 2021), and held a final participatory workshop to share findings from this comparative case study (Winter 2023). Between 30 and 50 people attended each of these activities. While not part of the data collection *per se*, this contributed to the quality of our study with regard worthiness of the topic, rich rigor and credibility (Tracy, 2010). Rigor was further supported by having two research assistants code and analyse the data independently before regrouping and comparing, regularly discussing coding and themes with the principal investigator and consulting co-investigators on final results interpretation. Involvement of a wide range of participants also meant information sources could be triangulated.

## RESULTS

**Fig. 1:**
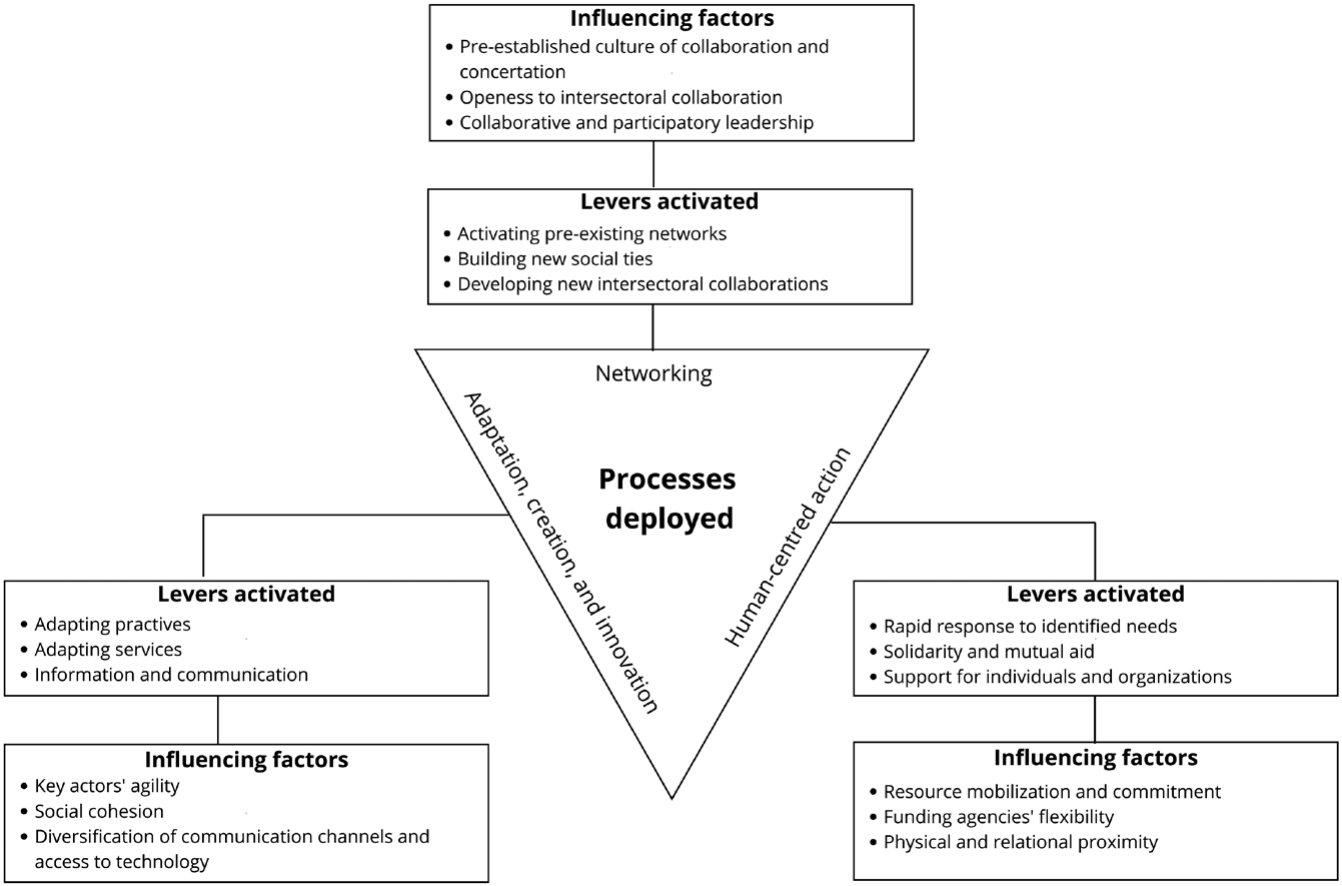
Processes deployed, levers activated and influencing factors associated with intersectoral collaboration and actions in favour of equity.

We identified three processes which communities deployed to support intersectoral collaboration and equity-focused action in the first wave of COVID-19 in Estrie: (1) networking; (2) adaptation, creation and innovation; and (3) human-centred action. As illustrated in Figure 1, different levers were used to activate each process, and several influencing factors facilitated or limited this activation. From ‘process’ to ‘lever’ to ‘influencing factor’, we move from the general to the specific, with influencing factors representing concrete elements upon which communities could act to develop or strengthen their resilience.

### Process 1: ‘networking’

The first process, ‘networking’, proved essential for supporting intersectoral collaboration and equity-focused action. Networking was enacted through three levers: activating pre-existing networks, building new social ties and developing new intersectoral collaborations. Activating pre-existing networks refers to the ability of actors involved in the COVID-19 response to strengthen relationships, intensify activities between collaborators and change the collaboration culture when necessary, for example, by welcoming new members to a concertation. The creation of a sanitary station for homeless people in Sherbrooke is a good example of this lever:

We created a [sanitary] station in the courtyard of [the non-profit] *Partage Saint-François* with the Sherbrooke Concertation Table on Homelessness. Everyone did what was needed according to their mandates: finding and setting up tents, recruiting workers, planning schedules… working at all levels in complementarity. [P2 – Sherbrooke]

Building new social ties refers to the ability of local institutions and formal actors to establish relationships with citizens. Although this, at times, required them to change roles and act more informally in relation to citizens, it was essential for compensating for the erosion of social interactions between citizens due to strict public health restrictions. This lever is exemplified by Granit MRC firefighters and police officers’ mobilization to show support towards hospital staff and elderly people living in long-term care homes. Developing new intersectoral collaborations refers to the creation of social infrastructures or collective initiatives enabling actors from different sectors who were not accustomed to working together to voluntarily do so with the aim of achieving a common goal. One example is the creation of crisis units such as the Community Action and Support Committee (*Comité d’entraide communautaire*) in Sherbrooke. In this case, co-coordination by a tandem of institutional and community actors and clear definitions of each partner’s mandate and role enabled the crisis unit to operate smoothly.

#### Influencing factors

Several factors were deemed to influence networking, either positively or negatively. We found this process to be facilitated by a pre-established culture of collaboration and concertation characterized by mature social infrastructures, openness to intersectoral collaboration, and reliance on collaborative and participatory leadership. Regarding the first factor, in some territories, social infrastructures created during previous crises, such as in the Sources and Granit MRCs, facilitated the energization of teams already experienced in crisis management who were able to get to work quickly to support the community. For example, the permanent outreach teams (*équipes de proximité*) created in the wake of the fatal 2013 derailment of an oil-carrying train in Lac Mégantic town centre were central to the COVID-19 response in the Granit MRC. In 2013, these multidisciplinary teams composed of health professionals, social workers, outreach workers and community organizers were tasked to deliver psychosocial services directly to the population, on the ground rather than in formal clinics, and were driven by principles of empowerment, inter-organizational and intersectoral collaboration, and citizen involvement and inclusion (Généreux *et al*., 2020). Similarly, existing ties also facilitated collaboration in the Haut-Saint-François territory where ‘A special COVID committee was created at the MRC level, whose members were already partners, they were already working together’ [P8 – Haut-Saint-François]. It was pointed out, however, that networking in this territory was nevertheless complicated by certain organizations’ strong culture of working in sectoral silos.

Openness to intersectoral collaboration is a second factor that facilitated networking. As one des Sources participant put it: ‘Social development is no longer just the business of the CDC [Community development corporation]. The MRC has a role and responsibilities’ [P3 – des Sources]. This new and heightened awareness of the importance of intersectoral collaboration helped mobilize a range of actors, such as outreach workers, rural agents, elected officials and citizen leaders, to actively participate in the COVID-19 response. For example, the synergy between actors involved in the Haut-Saint-François crisis unit and those on Sherbrooke’s Community Action and Support Committee made it easier to identify, reach and meet the needs of vulnerable groups, as did the adoption of a bottom-up approach, anchored in the field, rather than a top-down hierarchical one. On the flip side, some actors may not have been entirely open to intersectoral collaboration due to COVID-19 repercussions on themselves and their teams, such as exhaustion, demotivation and feelings of powerlessness, which limited their working across sectors.

Lastly, reliance on collective, participatory leadership is a third factor that could facilitate or hinder networking. Defined as leadership that recognizes skill complementarity, respects different partners’ values and organizational cultures, and supports the participation of all, this type of leadership generally enables a concerted response to community needs without duplication (World Health Organization, 2016). In this sense, the optimal sharing of responsibilities favoured the effective and coordinated contribution of actors with different fields of competence and expertise:

We all understood that it wasn’t every person for herself that would get us there, so we quickly got together virtually, to make our strategy together... we went around the table to get everyone’s opinion, be it community organizers, cultural actors, the school or municipality. [P8 – des Sources]

The collective leadership of three of the main players in the Québec community development ecosystem—the Health and Social Services Network, MRCs and Community Development Corporations—also enabled broad participation by various actors in crisis and monitoring units, despite the cumbersome nature of the process resulting from opposing modes of governance. While this was viewed positively in all territories, several participants nevertheless pointed out that at times, the Health and Social Services Network adopted more of a top-down leadership approach, delegating responsibilities without granting additional resources to take action, which compromised intersectoral actors’ mobilization. Furthermore, exclusion of certain key actors (e.g. minority linguistic groups) from consultations, local leaders’ lack of experience in crisis management and the late realization by some municipalities of their responsibility in helping manage the health crisis may also have hindered proper concerted and intersectoral response to the health crisis.

### Process 2: ‘adaptation, creation and innovation’

The second process we identified and through which intersectoral collaboration and equity-focused action were deployed was adaptation, creation and innovation. The three levers associated with this process related to adapting or innovating with regards practices, services and communications (Figure 1). Adapting practices involves adjusting ways of working and redeploying resources according to the evolving pandemic context (e.g. moving from meeting face-to-face to relying on virtual or hybrid modes) and needs (e.g. adjusting action plans according to emerging issues). Adapting services refers to organizations aligning their activities and services with the evolving context, for example by revising their mission or mandate to allow them to address emerging issues, as evoked by this participant:

Our mandate was not to communicate with the population, but to foster networking between organizations. But along the way, we saw the need to adjust. We set up a Facebook page to get in touch with people quickly. The information came from organizations. We also created an email address to redirect people to resources. [P1 – Sherbrooke]

In a related vein, adapting communications refers to diversifying the means deployed to promote access to reliable information at the right time as well as strategies aimed at promoting social cohesion through the maintenance of social interactions.

#### Influencing factors

We identified three factors which influenced the process of adaptation, creation and innovation: key actors’ agility, social cohesion, and diversification of communication channels and access to technology. First, key actors’ agility and flexibility in changing roles was found to facilitate adaptation, creation and innovation. One illustration of this lies in municipal authorities’ openness to step outside their usual sphere of influence and engage directly with higher levels of government. Adopting a positive and proactive attitude towards the pandemic reality also enabled them to demonstrate autonomy and leadership in crisis management. On the other hand, the centralization of powers within the State and public health authorities sometimes limited municipalities’ capacity for action, despite their openness to act. For example, while municipal bodies were responsible for overseeing the application of public health directives at the local level (e.g. enforcing the ban on indoor gatherings), uncertainties, controversies and conspiracy-thinking surrounding State-imposed health measures may have undermined public confidence in local institutions. This links back to the above discussion concerning the problematic discrepancy between the increased responsibility given by the State to municipal and other local actors on one hand, and the lack of resources and support provided to enact these responsibilities, on the other hand.

Key actors’ agility is further illustrated by the design and deployment of continuity plans structured according to the evolving reality and needs. While some private and third sector actors struggled with the new reality of high staff turnover, others were able to launch new services, products and programs to address emerging needs, making it possible to remedy certain blind spots and to ‘take care of everyone’. To do so, some were forced to step outside their usual field of intervention or redirect their attention to specific groups, as stated by this Sherbrooke participant from a third-sector organization:

It’s not part of our mission to help the most vulnerable people; we support social economy enterprises that can support vulnerable people directly. We’ve been more attentive to these businesses so that they stay in operation as much as possible. [P9 – Sherbrooke]

A second factor which facilitated adaptation, creation and innovation was social cohesion within communities, which is characterized by strong human ties and a benevolent approach between citizens and organizational actors (Manca, 2014). In this sense, adaptation to the pandemic context proved easier in territories where social and economic disparities were narrower and where community members shared common values and a strong sense of belonging. Adaptation to constantly changing public health orders also appeared to be less complex in rural areas, particularly those where the social, community, economic and physical environment was more conducive to interaction. This was also true in micro-territories (where everyone knew everyone else) and in sparsely populated but well-serviced areas (where citizens were more reachable). For example, the small geographical size, low population and high sense of belonging in the MRC des Sources meant that the needs of vulnerable groups could be managed on an almost case-by-case basis.

A third and final factor influencing adaptation, creation and innovation was the diversification of communication channels and access to technology. Communication indeed proved particularly challenging in the context of reduced physical and social interactions and heightened misinformation during COVID-19. According to participants, actors quickly realized they had to ‘to leave no stone unturned’ [P7 – des Sources] and communicate with citizens through more diverse channels (including in person, print, radio, TV and social media), more often than usual, and more simply to increase reach and reduce misinformation. The centralization of information and requests in the form of a specialized help desk (Granit), telephone line (des Sources) or newsletter (Sherbrooke) also enabled actors involved in the COVID-19 response to maintain a high degree of consistency in communications despite how quickly new information emerged. In the Sources and Sherbrooke MRCs, the Community Development Corporations led these initiatives, which, in addition to informing citizens of available services in a timely fashion also freed community organizations from this task. Reliance on outreach workers with established and close ties with citizens also enabled reaching more vulnerable groups. Obstacles to effective communication nevertheless remained, including frequent changes in directives coming from public health authorities and delays in processing, translating and disseminating information. Increased reliance on digital technology for working, learning and communicating with family also constrained adaptation, creation and innovation. Interestingly, this constraint was equally felt in urban and rural territories, but for different reasons. In the case of Sherbrooke, a lack of modern equipment was most problematic, and eventually solved by distributing second-hand computers to community organization employees working from home, while in rural territories, access to a high-speed internet network was most problematic. Although the turn towards virtual communication was positively referred to as ‘the greatest adaptation’ [P1 – Granit], over time, difficulties nevertheless became apparent, such as sustaining participants’ attention, motivation and commitment in virtual meetings, highlighting the limits of this adaptation in the longer term: ‘People are no longer able to do *visio*, they need to see each other’ [P1 – Haut-Saint-François].

### Process 3: ‘human-centred action’

Human-centred action is the third and final process through which intersectoral collaboration and equity were deployed in response to the first wave of COVID-19 in Estrie. These are defined as actions in which individuals, and particularly vulnerable groups and organizations, were the focal point. The three levers which supported this process are rapid response, solidarity and mutual aid, and support for individuals and organizations (Figure 1). Rapid response refers to key actors having to make prompt efforts to identify and respond to the general population and vulnerable groups’ needs, within the limits of available resources. For example, in the MRC des Sources:

Some high school students didn’t have Internet access at home. The town sent a request to the MRC and within 2 weeks, the students received USB keys to connect to the Internet..... We did it because it’s a priority for our young people, to fight against dropping out of school. [P3 – des Sources]

Solidarity and mutual aid refer to mutual assistance based on the principles of reciprocity and/or free help to the community. Examples of such actions include benevolent measures towards vulnerable groups, such as sending virtual cards and flowers to the elderly, making friendly calls to isolated people, and distributing food to families, as well as community organizations helping each other out by pooling their resources. Finally, support for individuals includes measures taken to reduce the consequences of poor living conditions on the health of vulnerable individuals and families (e.g. help with food shopping for people without social support), while support for organizations was actualized in the form of support for the functioning, operating and liquidity of organizations facing difficulties, as illustrated by a partnership with the Quebec entrepreneurial Coaching Clinic to offer targeted coaching to adapt to COVID-19 a support measure mentioned by a Granit participant.

#### Influencing factors

The following factors may have influenced the deployment of human-centred actions: resource mobilization, funding agencies’ flexibility, and physical and relational proximity. Most importantly, the mobilization and commitment of human, organizational, institutional, collective, financial, material and logistical resources in a spirit of mutual aid and solidarity facilitated this process. Resource mobilization was deemed easier in areas characterized by material or social fragility and by the presence of under-represented groups (e.g. linguistic, cultural and ethnic communities) as in Sherbrooke, potentially because there already were services dedicated to these more vulnerable groups. This was also deemed easier in small areas like the MRC des Sources and in areas with a self-reported ‘well-established reflex to help’ such as the MRC Haut-Saint-François. There, the spontaneous outpouring of individual support led the intersectoral group *Haut-Saint-François fou de ses enfants* to support the local food bank and volunteer centre in delivering food baskets and groceries. Despite widespread job and income losses linked to the pandemic, collective awareness of its impact on community organizations’ financial resources further encouraged donor generosity and resource mobilization in all territories: in des Sources and Granit MRCs, meals were delivered to seniors’ homes to ensure food security, while in Sherbrooke, restauranteurs donated their food stocks to community organizations.

Throughout the first wave of COVID-19, funding organizations also showed flexibility by maintaining subsidies to organizations despite the cancellation of planned events, while financial institutions introduced moratoria to facilitate loans and mortgage repayment which benefited individuals, organizations and businesses. This flexibility is both an example and enabler of human-centred action. However, participants from all territories mentioned that the federal government’s underfunding of community organizations hampered the deployment of human-centred actions through intersectoral collaboration since these organizations consequently faced important challenges, such as having to limit their activities or close temporarily due to resource shortages. Funding agencies’ flexibility and generosity was thus unequal, depending on the level of government represented. Finally, we found that physical and relational proximity contributed positively to human-centred action. Physical proximity between organizations which were ‘are all under the same roof, used to coordinating and seeing each other’ [P4 – des Sources] facilitated communication between intersectoral actors as well as the sharing of resources and deployment of concerted, rather than siloed, action. In a related vein, according to participants, relational proximity, which was fostered by a sense of belonging among urban citizens to neighbourhood tables and among rural citizens to local community groups, further made it easier to reach vulnerable groups and address their needs. This last influencing factor relates back to the processes of networking (e.g. through enabling activation of pre-existing networks) and to adaptation, creation and innovation (e.g. through facilitating communication).

## DISCUSSION

In this paper, we set out to identify the processes by which two interrelated dimensions of community resilience—intersectoral collaboration and equity-focused action—were deployed during the first wave of COVID-19 in Estrie, Québec, and to document factors facilitating or constraining these processes. We identified three processes: (1) networking; (2) adaptation, creation and innovation; and (3) human-centred action. For each process, we documented three levers that enabled their deployment, and three influencing factors. It has previously been suggested that intersectoral collaboration is essential to the design of a just and sustainable future (Ndumbe-Eyoh *et al*., 2021; Corbin, 2023), and it has been found to be central to an equitable response to COVID-19 (Public Health Agency of Canada, 2020). Our results align with the 2023 Canadian Public Health Officer’s report with regard to the importance of social infrastructure, intersectoral collaboration and a focus on more vulnerable groups for effectively and collectively responding to crises and emergencies (Public Health Agency of Canada, 2023). They further contribute to a better understanding of what can facilitate or hinder community resilience, and point to recommendations as to how communities can better prepare for future crises, whether health, social, economic or environmental.

Regarding networking, our results align with existing conceptual frameworks (Norris *et al*., 2008; Magis, 2010; Berkes and Ross, 2013) and findings from previous empirical studies (Nespeca *et al*., 2020; Slingerland *et al*., 2023). They highlight that ties established between intersectoral actors ahead of COVID-19, openness to intersectoral collaboration, and collective, participatory leadership all favoured an intersectoral and equity-focused response to COVID-19. They revealed that a well-established culture of collaboration and concertation characterized by mature social infrastructures can facilitate spontaneous collective action in a context of vulnerability, as reported by others (Rippon *et al*., 2020; Richard *et al*., 2023; Slingerland *et al*., 2023). For example, Richard *et al*. (Richard *et al*., 2023) found that networks pre-existing the pandemic were key to mobilizing actors involved in deploying government-led screening and contact tracing strategies in France. We further found that for actors otherwise accustomed to working in silos, an open-mind to intersectoral collaboration led to a growing awareness of its importance in responding to complex issues such as COVID-19. This suggests that intersectoral collaboration is possible in times of uncertainty, even in contexts where community development actors may be new to it.

Our findings regarding networking also stressed the need to ensure that observations from the field percolate up to higher levels of governance for meaningful and equity-focused action to be taken. In the context of a health crisis, this would mean creating networks that allow citizens, community organizations and institutions to work jointly, which can be facilitated by collaborative governance. In Estrie, this was embodied by the tripartite participatory leadership exercised by the Health and Social Services Network, MRCs and Community Development Corporations, which, although generally beneficial, nonetheless faced challenges related to power and resource imbalances. Similar challenges have been reported by Adger (Adger, 2003) according to whom effective networking in support of vulnerable groups requires collaborators to be involved on equal footing, by Morgan *et al*. (Morgan *et al*., 2021), who highlight the challenges of translating local voices into institutional change, and by Richard *et al*. (Richard *et al*., 2023), who recognize the complexity of developing intersectoral partnerships due to misunderstandings between different professions. Despite challenges, leaning towards collective and participatory governance where community organizations are allowed a seat at the table is promising. As discussed by Morgan *et al*. (Morgan *et al*., 2021), as coordinating entities of many different sectors, community organizations are indeed well positioned to develop and strengthen the systems and processes that enable citizens and institutions to work well together.

With regard the second process we identified—adaptation, creation and innovation—we found it to be fostered by key actors’ agility, social cohesion, and diversification of communication channels and access to technology. The ability to adapt and evolve is in fact one of the key strengths of a resilient community [adaptation being a defining characteristic of the phenomenon (Norris *et al*., 2008; Magis, 2010)] and others have found that it requires agility on the part of actors to change roles in times of crisis (Nespeca *et al*., 2020; Rippon *et al*., 2020; Slingerland *et al*., 2023). In our study as elsewhere (Badji *et al*., 2023; Slingerland *et al*., 2023), ease of adaptation was tied to the process of networking wherein, for example, formal actors such as firefighters adopted more informal roles in support of local hospital staff and elderly groups. We also found this process to be easier in places where social cohesion and sense of belonging were stronger. This resonates with previous literature documenting how a sense of shared values, interdependency and community could greatly foster commitment to sustained collective efforts in the face of COVID-19 (Wolf *et al*., 2020) as well as chronic ‘ordinary’ stressors such as sustained high levels of unemployment and poverty (Rochira *et al*., 2023). Similar to Comes (Comes, 2016), we also found that centralizing information and *communicating effectively*, *more often than usual*, and *using different media* was essential to reach a broad population and respond to challenges in the context of uncertainty and rapid change resulting from COVID-19. This aligns with the Institut National de Santé Publique du Québec’s recommendations (Institut National de Santé Publique du Québec, 2020) stating that disseminating information to promote community resilience requires mobilizing intersectoral partners and local opinion leaders and demonstrating empathy to build public trust. Our results also revealed the essential nature—but inequitable distribution—of high-speed Internet and digital literacy. As noted by Ndumbe-Eyoh *et al*. (Ndumbe-Eyoh *et al*., 2021), limited access to the Internet may prevent certain groups (e.g. those on low incomes or the elderly) from taking part in the virtual turn. While we found this to be a barrier to reaching specific groups and to service continuity in some territories, most were nevertheless able to overcome this challenge by tapping into collective generosity through donations of mobile internet USB keys to students and second-hand computers to those in need.

The last process we documented, human-centred action, was found to be influenced by resource mobilization, funding agencies’ flexibility, and physical and relational proximity. We found mobilizing resources to be easier in communities that were already fragile and/or had a strong and established culture of mutual aid. This could be explained by such territories already having services dedicated to more vulnerable groups and being used to relying on their own local infrastructures and social networks to solve problems too often neglected by public health and emergency response systems. As pointed out by Rochira *et al*. (Rochira *et al*., 2023), communities’ potential to respond effectively to crises depends on their access to diverse resources, but also on their ability to make available resources (even when minimal) work together. Our results also suggest that collective action geared towards vulnerable groups was facilitated by the physical and relational proximity of intersectoral actors. Morgan *et al*. (Morgan *et al*., 2021) similarly reported that in Toronto, Canada, local community leaders who were close to their clientele played a key role in mitigating the psychosocial and socioeconomic impacts of the pandemic by responding quickly to their fellow citizens’ needs in terms of food provision, information and mental health support. Regarding relational proximity, Ndumbe-Eyoh *et al.* (Ndumbe-Eyoh *et al.*, 2021) suggest the community sector is well positioned to provide services and support in times of urgency, but that it should not be given full responsibility for filling the gaps in the public health and emergency management system (Morgan *et al*., 2021; Ndumbe-Eyoh *et al*., 2021). This may be especially true when the community sector is not provided with all the resources to do so (den Broeder *et al*., 2022), as we found here. Efforts should therefore be expended to ensure equity in power, responsibilities and resources across intersectoral actors involved in emergency responses.

### Strengths and limitations

Our study has several strengths, the main one being the participatory approach we adopted to respond to practical concerns expressed by community development actors. An interdisciplinary and intersectoral advisory committee composed of researchers and representatives from the non-profit sector, health and social services and regional governments also developed data collection procedures and tools, and provided insights throughout the study. We led workshops with key actors from each territory at several time points which allowed us to gain feedback on preliminary and final results. Our participatory research process was highly mobilizing itself and had the potential to support reflexivity, autonomy and learning among participating communities with regard their territory’s resilience (Ross and Berkes, 2014), something which was also reported by participants. Although only four of the nine Estrie territories were represented in this paper, we can confidently state that the results reflect the diversity of realities lived in the region. Moreover, our territorial approach aligns with the United Nations Sendai Framework (United Nations Office for Disaster Risk Reduction, n.d.), the ‘One Community at a time’ approach to resilience (Ma *et al*., 2023), and the recent Canadian Chief Public Health Officer’s report on resilience to future crises (Public Health Agency of Canada, 2023), which all stress the importance of learning from local communities to then share lessons more widely to collectively seek to ‘build back better’, which is what we aimed for here.

Our study has limitations, one being that it focused on the first wave of COVID-19. While this testifies to our responsiveness, the pandemic eventually lasted several months, and the processes deployed to support community resilience may have evolved over time. Despite this, the lessons learned at the start of the crisis, which characterize communities’ response at the height of an emergency situation when there was a great deal of uncertainty, can guide resilience actors in better and more quickly responding to future crises. Still, it would have been interesting to explore how intersectoral collaboration and equity-focused action evolved over different waves of COVID-19 and to assess the extent to which the processes and levers documented here could be deployed on a perennial basis. Finally, although we discussed the three processes independently for ease of presentation, we should highlight that they were, to some extent, interconnected. For instance, it could be easier to be creative in deploying human-centred actions if strong networks are in place, and this should be kept in mind when interpreting our study results.

## CONCLUSION

Intersectoral collaboration and equity are central to community resilience in general, and in hindsight, they also proved essential to an effective and equitable response to COVID-19. We identified three processes—networking; adaptation, creation and innovation; and human-centred action—which favoured the deployment of intersectoral actions in favour of equity and vulnerable groups during COVID-19 by Estrie communities. Although our study focused on a single region of Quebec during the first wave of the pandemic, it contributes to a better understanding of what may facilitate or hinder the development and strengthening of community resilience in times of crisis, in both urban and rural contexts. Since local communities are at the forefront of crises when they occur, they feel their effects immediately and directly and should therefore also be at the heart of response and recovery. In this vein, our findings suggest ways in which communities can improve or build upon assets and strengths developed during the pandemic to better prepare for future crises, be they health, social, economic or environmental.

## Data Availability

The data underlying this article will be shared on reasonable request to the corresponding author.
